# Role of textural heterogeneity parameters in patient selection for 177Lu-PSMA therapy via response prediction

**DOI:** 10.18632/oncotarget.26051

**Published:** 2018-09-07

**Authors:** Zain Khurshid, Hojjat Ahmadzadehfar, Florian C. Gaertner, László Papp, Norbert Zsóter, Markus Essler, Ralph A. Bundschuh

**Affiliations:** ^1^ Department of Nuclear Medicine, University Hospital Bonn, Bonn, Germany; ^2^ Center for Medical Physics and Biomedical Engineering, Medical University of Vienna, Vienna, Austria; ^3^ Mediso Medical Imaging Systems, Budapest, Hungary

**Keywords:** ^177^Lu-PSMA therapy, ^68^Ga-PSMA, prostate cancer, tumor textural heterogeneity, response prediction

## Abstract

**Purpose:**

Prostate cancer is most common tumor in men causing significant patient mortality and morbidity. In newer diagnostic/therapeutic agents PSMA linked ones are specifically important. Analysis of textural heterogeneity parameters is associated with determination of innately aggressive and therapy resistant cell lines thus emphasizing their importance in therapy planning. The objective of current study was to assess predictive ability of tumor textural heterogeneity parameters from baseline ^68^Ga-PSMA PET prior to ^177^Lu-PSMA therapy.

**Results:**

Entropy showed a negative correlation (r_s_ = −0.327, *p* = 0.006, AUC = 0.695) and homogeneity showed a positive correlation (r_s_ = 0.315, *p* = 0.008, AUC = 0.683) with change in pre and post therapy PSA levels.

**Conclusions:**

Study showed potential for response prediction through baseline PET scan using textural features. It suggested that increase in heterogeneity of PSMA expression seems to be associated with an increased response to PSMA radionuclide therapy.

**Materials and Methods:**

Retrospective analysis of 70 patients was performed. All patients had metastatic prostate cancer and were planned to undergo ^177^Lu-PSMA therapy. Pre-therapeutic ^68^Ga- PSMA PET scans were used for analysis. 3D volumes (VOIs) of 3 lesions each in bones and lymph nodes were manually delineated in static PET images. Five PET based textural heterogeneity parameters (COV, entropy, homogeneity, contrast, size variation) were determined. Results obtained were then compared with clinical parameters including pre and post therapy PSA, alkaline phosphate, bone specific alkaline phosphate levels and ECOG criteria. Spearman correlation was used to determine statistical dependence among variables. ROC analysis was performed to estimate the optimal cutoff value and AUC.

## INTRODUCTION

Prostate cancer being the most common cancer and second leading cause of death among men in the Western world [1] is under constant surveillance by researchers and medical personnel worldwide. Despite the efforts, prostate cancer tends to be highly aggressive and can lead to significant mortality and morbidity [2]. It is of utmost importance to devise new methods aiming for earlier diagnosis and optimum individualized therapy of prostate cancer.

Among the ongoing advancements for the treatment of prostate cancer the possibilities involving Prostate specific Membrane Antigen (PSMA) have gained momentum. In the light of published data it can be deduced that radioligand therapy (RLT) with ^177^Lu-PSMA is effective and has a low toxicity profile [3]. It is also observed that up to 30% of patients do not show and prostate specific antigen (PSA) decline in response to RLT [3, 4]. Therefore, establishment of pre-therapeutic biomarkers for patient selection for RLT would be helpful to avoid over-treatment.

However, there is a continuous strive for establishment of pre therapeutic parameters in order to have an earliest possible assessment to therapy response, which can in turn guide towards redesigning or modifications in treatment course, thus directly affecting the patient survival. It can also help in selection of patient groups that can have maximum benefit from therapy. So far, such biomarkers are not available. Only low platelet counts and permanent use of an analgesic medication for bone pain are negative predictors of therapy response [5]. In contrast, pre-therapeutic tracer uptake as determined by visual assessment or SUV-measurement does not predict therapy response.

In the recent years, tumor textural analysis (a measurement of spatial heterogeneity) as acquired through PET scans is becoming increasingly important for extracting useful information concerning response prediction. PET having the ability for physiological imaging can provide in-depth information about cell metabolism, perfusion, cell proliferation, morphology, tumor viability and receptor density [6]. All these factors can contribute towards tumor heterogeneity and can in turn predict the therapy response. In most cases, these parameters have been used for assessment between pre and post therapy PET scans. For example, several tumors including sarcoma [7], head and neck tumors [8], esophageal carcinoma [9], NSCLC [10], rectal carcinoma [11] have been analyzed this way. However, this information can be even more beneficial if acquired as early as possible in the course of therapy. Different attempts have been made to acquire relevant data in the earlier treatment stages and even from the pre-therapeutic diagnostic scans and/or clinical parameters [12]. Most of these efforts have been made utilizing the anatomical imaging such as MR imaging or CT. However, no large amount of data is available highlighting the use of baseline PET scans as response predictors or therapy planners with particular emphasis on the role of textural heterogeneity parameters in prostate cancer for patients undergoing PSMA therapy. A study which elaborated the predictive role of textural features to predict outcome from base line PET scan showed significant results in cervix and head and neck cancers [13] and one other predicting survival in sarcoma patients [7]. Combination of latest advancements like therapy with PSMA-ligand labels with ^177^Lu and analysis of tumor textural heterogeneity may have a potential to provide first-rate information. Especially as recent studies found a non-responder rate of about 30% (no PSA decline) after radiopeptide therapy with ^177^Lu-PSMA [5, 14–16].

The objective of this current study was hence to assess the predictive ability of tumor textural heterogeneity parameters from baseline ^68^Ga-PSMA PET scan. Selected textural heterogeneity parameters had been previously widely used in different studies and showed a significant potential for depicting the outcome [9–11, 17, 18]. The predictive value of these parameters was compared to established clinical parameters (PSA, serum and bone alkaline phosphate, Eastern Cooperative Oncology Group (ECOG) criterion).

## RESULTS

**Figure 1:**
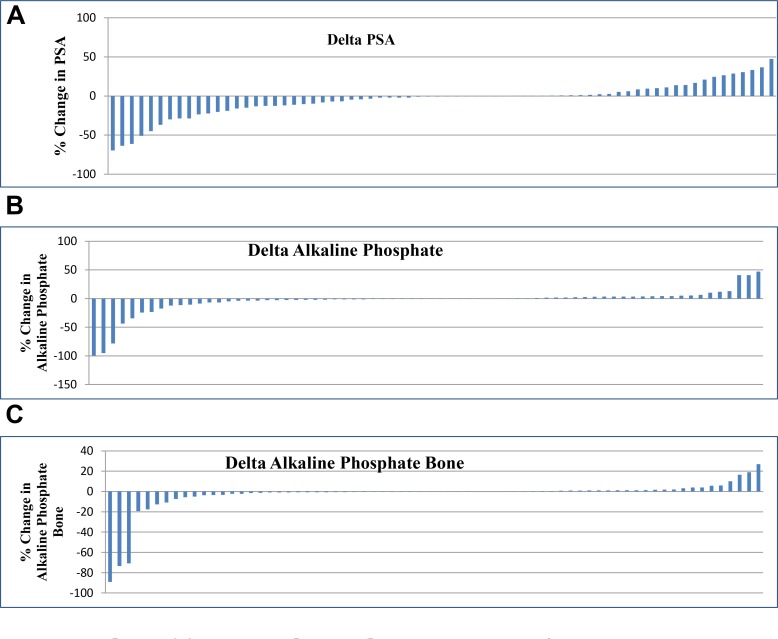
(**A**) Percentage change in PSA. (**B**) Percentage change in Alkaline Phosphate. (**C**) Percentage change in Alkaline Phosphate Bone.

Seventy patients were evaluated in this study. Decrease in PSA level was observed in 42/70 patients (60%) and they were labeled as responders to therapy. Increase in PSA level was seen in 28/70 patients (40%) considered as non-responders (Figure 1A).

41/70 patients (58%) showed response via decrease in serum alkaline phosphate level (Figure 1B) and 39/70 patients (55%) showed response by decrease in bone specific alkaline phosphate (Figure 1C). Among the responders 24/70 patients (34.2%) showed decrease in both PSA and alkaline phosphate levels at the same time while 22/70 patients (31.42%) showed decrease in PSA, alkaline phosphate and bone specific alkaline phosphate levels together. No change was observed in ECOG of any patient.

Analysis of PET based heterogeneity parameters revealed that only two textural heterogeneity parameters entropy and homogeneity showed correlation with change in pre and post therapy PSA levels. Textural heterogeneity parameters which showed correlation with PSA were derived from bone lesions. Parameters obtained from lymph node and other lesions did not show any correlation with any of correlating clinical parameters. Change in pre and post therapy values of serum alkaline phosphate, bone specific alkaline phosphate and individual patient ECOG status derived from all types of lesions remained uncorrelated. Similarly textural heterogeneity parameters other than entropy and homogeneity also remained uncorrelated. Actual values of correlating parameters as obtained through Spearman correlation are as under.

**Figure 2:**
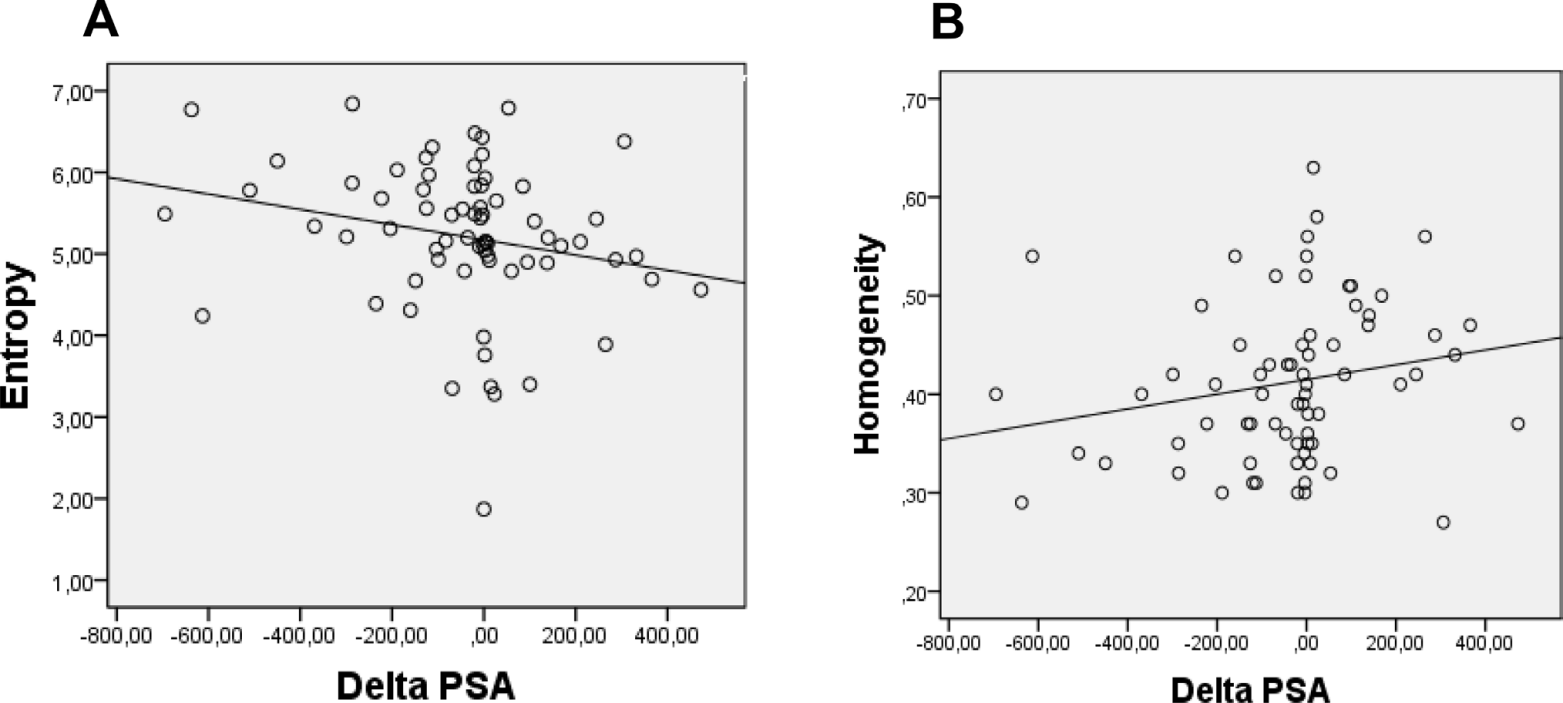
(**A**) Representation of negative correlation between absolute ΔPSA(ng/ml) and entropy of bone lesions (R^2^ = 0.283). (**B**) Representation of positive correlation between absolute ΔPSA (ng/ml) and homogeneity of bone lesions (R^2^ = 0.326).

Entropy showed a negative correlation (r_s_ = −0.327 and *p* = 0.006) and homogeneity showed a positive correlation (r_s_ = 0.315 and *p* = 0.008) with change in pre and post therapy PSA levels (Figure 2A and 2B). It is essential to be taken into account that change in PSA levels was obtained as post therapy levels minus the pre therapy level (post therapy PSA–pre therapy PSA). As described in methods a negative value of this equation meant that post therapy PSA was less than that of pre therapy and the case was considered as of a responder. So the resultant value of a responder was negative and vice versa. Hence, a negative correlation of entropy with this change (also represented with a negative value) meant that entropy and change in PSA levels was directly proportional to each other. Or in other words the responders showed a higher entropy value. Similarly, homogeneity showed a positive correlation and hence a negative proportionality with the change in PSA levels signifying that the responders had a lower homogeneity.

Table 1 summarizes the positive results i.e. obtained by correlating positive textural heterogeneity parameters obtained from bone lesions with change in pre and post therapy PSA levels. Rest of the parameters are also given for comparison. It can be also seen that SUV values also did not positively correlate.

**Table 1:** Correlation of bone lesion derived PET parameters with change in PSA level

PET parameter (Bone lesions)	Correlating clinical parameter	Spearman coefficient	*p*-value
Entropy	ΔPSA	0.327	0.006
Homogeneity	ΔPSA	–0.315	0.008
COV	ΔPSA	0.113	0.516
Contrast	ΔPSA	0.257	0.136
Size Variation	ΔPSA	–0.309	0.071
SUV(mean)	ΔPSA	0.168	0.333

The ROC analysis also showed that entropy and homogeneity are statistically significant (*p* < 0.05) for predictive ability of pre-therapeutic PET. Further results of ROC analysis are summarized in the Table 2.

**Table 2:** Results of ROC analysis for predictive value of pre therapeutic PET-CT

Parameter	AUC (Area under curve)	95% Confidence interval	Cut-off value (based on youden index)
Entropy	0.695	0.57 to 0.799	>5.15
Homogeneity	0.683	0.56 to 0.789	≤0.43

**Figure 3:**
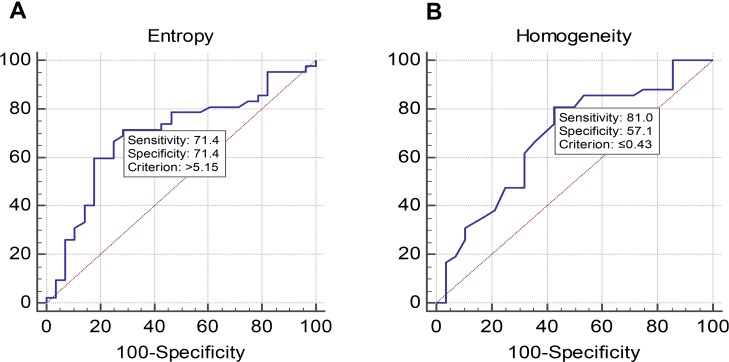
(**A**) and (**B**) Showing results of ROC analysis.

Sensitivity and specificity was also assessed for both parameters through ROC (Figure 3A and 3B) and these values were used to find out positive and negative predictive values (Table 3).

**Table 3:** Outcomes of positive parameters

Parameter	Sensitivity	Specificity	Positive predictive value	Negative predictive value
Entropy	71.4%	71.4%	62.5%	78.9%
Homogeneity	81.0%	57.1%	66.6%	73.9%

For the above mentioned parameters, the combined sensitivity of entropy and homogeneity for predicting outcome was 57.83% and the combined specificity was 87.74%. The Pearson correlation and linear regression analysis did not show any dependence between textural heterogeneity parameters and lesion volume. The value of R for correlation between volume and entropy and volume and homogeneity was 0.002 and 0.004 respectively.

## DISCUSSION

In our study, we analyzed the predictive capability of textural heterogeneity parameters in patients undergoing ^177^Lu-PSMA therapy for determination of patient selection criteria, treatment outcome and survival analysis. The retrospective German multicenter analysis [15] showed that ^177^Lu-PSMA RLT demonstrated a PSA decline occurred in 65% of patients after 1 cycle of RLT. There are still almost 30% of the patients who did not show or showed less than 50% decline in serum PSA level. It is very important to identify those patients and so that therapy modifications might be performed which can then help in increasing the efficacy of treatment. Our study aimed at utilization of heterogeneity parameters in an effort to improve the selection criterion of patients and acted as a means to predict improved outcome. Our study showed a potential for response prediction through baseline PSMA PET-CT scan using textural features. It also suggested that more heterogeneous the tumor was in PSMA expression more responsive it was to PSMA therapy, thus contributing efficiently towards patient selection, treatment planning and improvement in overall diagnostic accuracy. The ROC analysis showed that two textural heterogeneity parameters entropy and homogeneity were statistically significant (*p* < 0.05) for predictive ability as obtained from the baseline ^68^Ga-PSMA scan prior to ^177^Lu-PSMA therapy. Spearman correlation showed that entropy showed a negative correlation (r_s_ = −0.327 and *p* = 0.006) and homogeneity showed a positive correlation (r_s_ = 0.315 and *p* = 0.008) with change in pre and post therapy PSA levels.

Predictive ability of various parameters from the baseline scan has also been previously investigated. Ferdinandus *et al.*, 2017 [5] analyzed the effect of different pretherapeutic parameters on the therapeutic response measured by prostate-specific antigen (PSA) 2 months after radioligandtherapy. Numerous studies have also reported the use of textural heterogeneity parameters for the assessment of patient outcome. Eary *et al.*, [7] proposed that heterogeneity in ^18^F-FDG spatial distribution can be used to predict tumor biologic aggressiveness. In another study by Cheng *et al.*, 2013 [8] the researchers showed that two textural heterogeneity parameters entropy and homogeneity showed ability to predict outcome in patients with advanced T-stage oropharyngeal squamous cell carcinoma. However, the conventional PET parameters SUV mean and max did not show such ability. Tumor volume also had no effect on textural heterogeneity.

Similarly in another study by Tixier *et al.*, 2011 [9] the aim was to evaluate textural analysis of baseline PET scans for the prediction of therapy response in esophageal cancer. Receiver-operating-characteristic curve analysis showed that tumor textural analysis can provide non-responder, partial-responder, and complete-responder patient identification with higher sensitivity (76%–92%) than any SUV measurement. Textural features of tumor metabolic distribution extracted from baseline ^18^F-FDG PET images allow for the best stratification of esophageal carcinoma patients in the context of therapy-response prediction. In our study the combined sensitivity of entropy and homogeneity for predicting outcome was 57.8% and the combined specificity was 87.7%.

It is interesting to note that in our results entropy showed a directly proportional correlation with change in pre and post therapy PSA levels while homogeneity showed an inverse relationship. In other words it can be inferred that more heterogeneous the tumor was, the better it responded to the PSMA therapy. As higher entropy is a measure of greater heterogeneity of the tumor. The patients which were labeled as responders owing to decrease in post PSMA therapy PSA levels showed a higher entropy in baseline scan. Similarly, the responders showed lower homogeneity in the baseline scan. Although we did define the response to therapy by PSA levels and did not correlate the textural features with patient outcomes, we have shown in previous studies, that a reduction of PSA after therapy with Lu-177 PSMA is a prognostic factor for overall survival [14, 19]. Therefore, we can assume that the PSMA expression on the tumor cell surface of patients included in the present study was quite high. With increasing heterogeneity, the PSMA expression increased, which might pave the way for an increased uptake of PSMA bound ligands. Consequently, it can be hypothesized that those tumors respond better to therapy. Hence, our study showed that with increased heterogeneity in PSMA expression, a better response to PSMA therapy could be expected.

In a study by Pyka T *et al.* [10] predictive value of textural heterogeneity parameters in FET-PET for recurrence and prognosis in non-small cell lung carcinoma (NSCLC) patients receiving primary stereotactic radiation therapy (SBRT). This study showed that entropy has predictive potential for local recurrence with an AUC of 0.872. The study also showed that higher value of entropy was linked to poor outcome. In our study entropy was also a predictor for outcome with an AUC of 0.695 however, higher entropy showed better outcome for PSMA therapy.

An interesting question which arises here is whether a more heterogeneous tumor can respond better to the treatment? In many previous studies involving textural heterogeneity it was proven otherwise. Increased textural heterogeneity has already been linked with poor outcome. On the contrary, our study points in the opposite direction. One of the reasons for this behavior could be that this phenomenon can possibly be highly tumor and therapy specific. PSMA shows significant over expression in metastatic, poorly differentiated and therapy refractory carcinomas. Treatment refractory tumors can have the presence of multiple clones resulting in formation of complex systems and contributing towards tumor heterogeneity [19]. Patients included in our study group had already metastatic disease which was treatment refractory. Therefore, we can assume that there was a significant PSMA overexpression in tumors of patients included in our study. More heterogeneous a tumor is, more PSMA expression it shows thereby increasing the uptake of PSMA bound ligands and thus responding better to therapy. In a very interesting study by Jeffrey West and Paul Newton [20] about optimizing chemo-scheduling based on tumor growth rates discussed ways to optimize chemotherapeutic scheduling using a Moran process evolutionary game-theory model of tumor growth that incorporates more general dynamical and evolutionary features of tumor cell kinetics. It proves the fact that over multiple cycles, higher entropy strategies have a bigger impact on faster growing tumors than on slower growing tumors.

Hence, firstly this study showed the possibility of extracting vital data via the analysis of baseline scan only which can directly predict the outcome of patient. This finding can be of excessive importance in selecting the patients which can possibly respond better by altering the treatment regimen. Secondly, this study differentiates the textural parameters which can be used for gaining outcome data and also points out their correlation with the outcome.

This study had some limitations. First, the reproducibility of tumor heterogeneity as assessed by PSMA PET-CT has not been much explored yet. For the assessment of this robustness repeated analysis of PSMA PET-CT studies will have to be performed in a short interval of time. For FDG-PET-CT, such a study has been performed by Tixier *et al.* and demonstrated reproducibility of textural parameters comparable to the range of conventional SUV [9]. They found that several textural parameters showed reproducibility comparable to the range of conventional SUV. Therefore, these parameters can be applied for therapy response assessment at least with the same confidence as SUV.

The analysis of heterogeneity can also be limited by the size of the lesion. If the lesion becomes too small, the analysis of differences in radiotracer uptake within the lesion does not make sense. Investigating small structures, e.g. lymph node metastases, may challenge the value of textural parameters. In our study, the smallest lesion was 7.8 cm^3^, which is still about 62 voxels. A second point that needs further investigation is the influence of reconstruction parameters on tissue heterogeneity. PET reconstruction algorithms require smoothing of the raw image data which could influence assessment of tumor textural heterogeneity. PET images assessed in this study were reconstructed using the standard protocols for clinical routine at our institution. For comparison of changes in tumor heterogeneity, all images were acquired and reconstructed with the same set of parameters. Metabolically active tumor volumes were delineated manually instead of using segmentation algorithms with fixed thresholds and might therefore be prone to interindividual differences. However, the appropriate segmentation method is still widely discussed; semiautomatic methods often fail depending on the tumor localization [21, 22]. Therefore, we considered manual delineation to be optimal for our study especially as we included metastases varying in location as well as signal-to-background ratio. Additionally, Hatt and colleagues [23] could demonstrate that the predictive value of textural parameters is not affected by partial volume effect and is relatively independent of the method used to delineate the tumor volumes to be analyzed.

## MATERIALS AND METHODS

### Inclusion criteria

70 patients with histologically proven prostate cancer were retrospectively included in this study. Clinical data was collected from November 2014 to April 2016. All patients were planned to undergo ^177^Lu-PSMA-617 (abbreviated as ^177^Lu-PSMA in this study) RLT. Average age of patients was 71.46 years. Inclusion criteria for this retrospective analysis were progressive metastatic castration-resistant prostate cancer (mCRPC) patients. Patients experienced progression under next-generation androgen-deprivation therapy (e.g., abiraterone, enzalutamide) or first- or second-line chemotherapy (e.g., docetaxel, cabazitaxel) or were not eligible for chemotherapy. All patients eligible for ^223^Ra received this treatment before undergoing ^177^Lu-PSMA RLT. 39 patients had prior chemotherapy. 16 patients had been treated previously with ^223^Ra, while 27 patients had previous external beam radiation therapy (EBRT). Patient characteristics are shown in Table 4.

**Table 4:** Patient characteristics

Characteristic	Data
Age	71.5 years (48–88 years)
**Site of metastasis:**	
Bone	70 (100%)
Lymph node	33 (47.1%)
Other (liver, prostate)	15 (21.4%)
**Previous therapy of mCRPC:**	
Androgen deprivation therapy	70 (100%)
Chemotherapy	39 (55.7%)
^223^Ra	16 (22.8%)
EBRT to bone	27 (38.5%)

### ^68^Ga-PSMA scan

A ^68^Ga-PSMA PET scan was performed for every patient. Each patient underwent ^68^Ga-PSMA scan before therapy with ^177^Lu-PSMA termed as the baseline scan. The objectives of the baseline scan included staging and therapy planning for all patients. After the baseline scan patients underwent ^177^Lu-PSMA radioligand therapy. Renal function of every patient was analyzed prior to therapy with ^99m^Tc-MAG3 renal scintigraphy.

Data were acquired with a Biograph Sensation 2 PET/computer tomography (PETCT) scanner (Siemens Medical Solutions). The axial and transverse fields of view were 16.2 and 58.5 cm respectively. The transverse resolution of the scanner was about 6.5 mm, whereas the axial resolution was 6.0 mm, both at a radius of 10 mm. The computer tomography (CT) component was a 2-slice spiral CT scanner. About 73 minutes (range 50–90 minutes) after the intravenous injection of approximately 131.3 MBq (range 98.8 to 174.8 MBq) of ^68^Ga-PSMA, the patient was placed in the scanner. Low dose CT from the head to mid-thighs was performed followed by the PET scan of the same area in 6–7 bed positions, each for 3–4 minutes depending on the body weight of the patient. The CT data were reconstructed in 512 × 512 pixel matrices. PET data was reconstructed into 128 × 128 matrices in axial, coronal and sagittal planes using the iterative attenuation-weighted ordered subset algorithm implemented by the manufacturer using 4 iterations and 16 subsets. Attenuation and scatter correction was performed using the CT data. Final voxel size was 5.3 mm × 5.3 mm × 5 mm. All patients gave written and informed consent to the imaging procedure. All patient record and information was anonymized before analysis.

### PET data analysis

**Figure 4:**
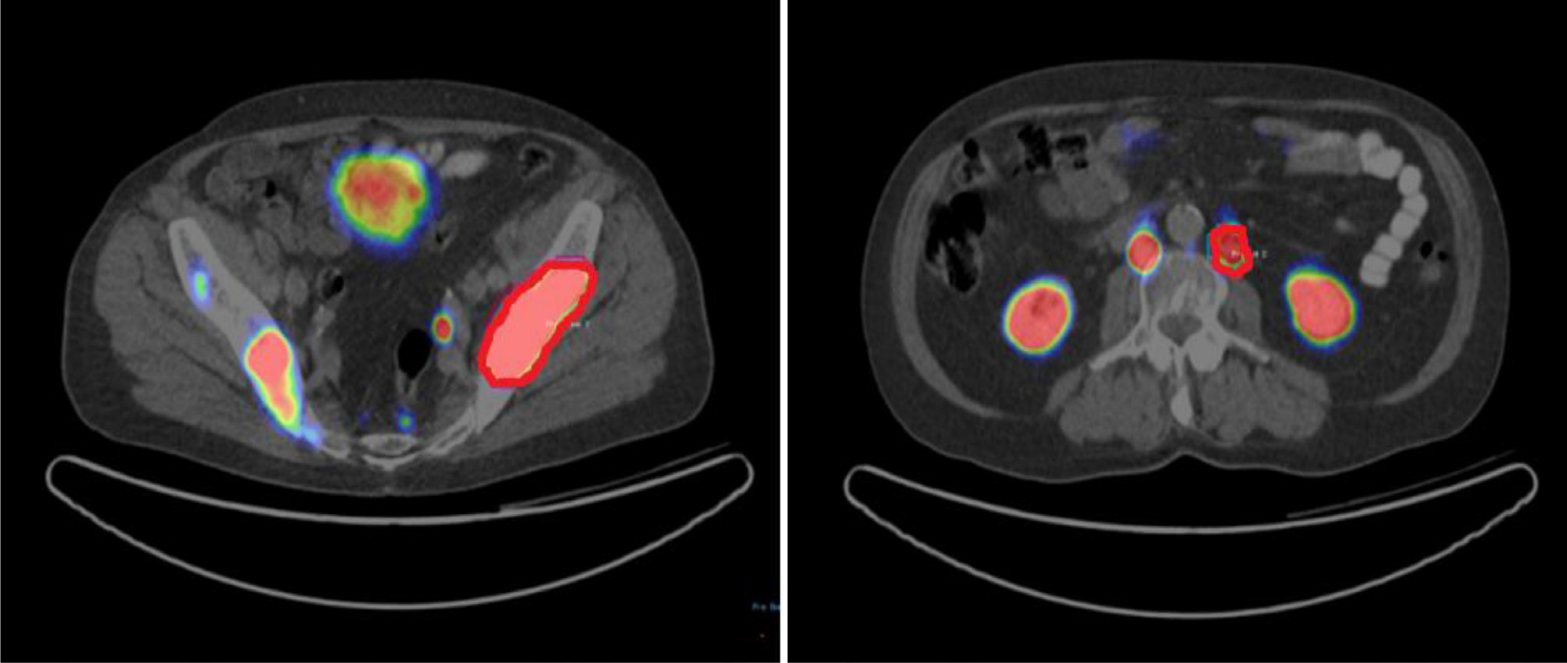
VOIs for analysis of bone and lymph node lesions

Image data were transferred to an Interview Fusion Workstation (Mediso Medical Imaging System, Budapest, Hungary). Firstly, co-registration between PET and CT images was performed. Tumor volume was manually delineated on PET images (Figure 4) with a standard uptake value (SUV) threshold [24, 25]. All the 70 patients had bone metastasis. Thirty three patients had lymph node metastasis along with bone metastasis. Fifteen patients had additional liver and/or prostate lesions. Three VOIs each for bone and lymph node lesions were delineated manually. Other lesions were also delineated if present in liver and prostate. Parameters to be evaluated were measured in these VOIs. A total of 328 VOIs were delineated. Mean volume of the lesions was 32.9 cm^3^ (range 7.8 cm^3^ to 82.3 cm^3^). For each patient three bone lesions were marked. Similarly, three lymph node and other (liver and prostate) lesions were delineated where applicable. For every patient on average six VOIs were marked (range 3–12 depending on organ involvement. For final analysis a mean value of every included parameter was determined.

Tumor textural heterogeneity was assessed by extraction of local and global textural features from uptake histogram analysis and normalized gray-level co-occurrence matrix (NGLCM) respectively [17]. The selected heterogeneity parameters were COV, entropy, homogeneity, contrast and size variation (Table 5).

**Table 5:** Overview of textural heterogeneity parameters

Parameter	Order	Description
COV	1st	A normalized measure of dispersion of a frequency distribution (standard deviation divided by the mean value of the activity concentration in the tumor volume).
Entropy	2nd	Measures randomness of distribution, e.g. a homogenous matrix demonstrates low entropy.
Homogeneity	2nd	A measure for continuous areas of same or similar voxel values in an image or voxel of interest (VOI).
Contrast	2nd	A measure of local variations present in the image. A high contrast value indicates a high degree of local variation.
Size Variation	3rd	Measures the difference of the grey value when going to the next voxel. It is high when the intensity changes very often between single voxels.

The selected parameters have been used widely in numerous PET studies and showed a statistically significant ability to depict the role of textural heterogeneity for analysis of tumor behavior [7–12, 17, 18]. SUV mean histogram analysis was used to calculate coefficient of variation (COV) [6, 26]. Rest of the parameters, entropy, homogeneity, contrast and size variation were calculated from NGLCM contained three dimensional gray-level information [6, 26]. For comparison purpose SUV mean as a conventional PET parameter was also analyzed.

### Treatment response

After the baseline scan all patients underwent ^177^Lu-PSMA therapy. The decision for ^177^Lu-PSMA radioligand therapy was made by the local interdisciplinary tumor board at each therapy center. The protocol followed for therapy has already been explained in detail by Rahabar *et al.* [15]. The parameters used to assess the response to ^177^Lu-PSMA therapy were pre and post therapy changes in levels of PSA (prostate specific antigen), serum and bone alkaline phosphate and Eastern Cooperative Oncology Group (ECOG) criterion. Time difference between pre and post therapy levels was 7.1 weeks (average 6–8 weeks). Change in levels of clinical parameters was obtained as post therapy levels minus the pre therapy level (post therapy PSA – pre therapy PSA). A negative value of this equation meant that post therapy PSA was less than that of pre therapy and the case was considered as of a responder and viceversa.

### Statistical analysis

The statistical analysis was performed using SPSS (version 22, IBM). To evaluate the correlation between conventional and textural heterogeneity parameters and changes in pre and post therapy clinical parameters Spearman correlation was used. Statistical tests were conducted at a two-sided level of significance as *p* < 0.05. A multivariate regression analysis was performed to see the dependence between textural heterogeneity parameters and the lesion volume.

Receiver-operating-characteristics (ROC) analysis was also performed using MedCalc software (version 12.3.0.0; MedCalc). ROC analysis was performed to estimate the optimal cutoff value of the correlating parameters for response assessment. For this purpose, the Youden index was used to maximize the sum of sensitivity and specificity [27]. The area under the curve (AUC) was calculated for each parameter using the nonparametric method developed by Hanley and McNeil [28] representing the overall predictive or prognostic performance. For AUCs, exact binominal confidence intervals were calculated (95% confidence level), indicating the statistical significance of predictive capability if the critical value of 0.5 is not included.

To see any correlation between lesion size and parameters with predictive capability Pearson correlation and linear regression analysis was performed.

## CONCLUSIONS

Our study showed a potential for response prediction for ^177^Lu-PSMA therapy through baseline PSMA PET-CT scan using textural features. It also suggested that more heterogeneous the tumor was in PSMA expression more responsive it was to PSMA therapy, thus contributing efficiently towards patient selection, treatment planning and improvement in overall diagnostic accuracy.
